# Children’s Perception of Parental Body Size and Its Association with Their Desire to Be Like the Parent

**DOI:** 10.3390/children13101404

**Published:** 2026-10-14

**Authors:** Abdullah Kaan Kurt, Serdar Karakullukçu

**Affiliations:** 1 Department of Family Medicine, Faculty of Medicine, Karadeniz Technical University, 61080 Trabzon, Türkiye; 2 Department of Public Health, Faculty of Medicine, Karadeniz Technical University, 61080 Trabzon, Türkiye; serdar.karakullukcu@ktu.edu.tr

**Keywords:** body image, body size, parent-child relations, child, pediatric obesity

## Abstract

**Highlights:**

**What are the main findings?**

- Parents showed moderate agreement, whereas children aged 4–8 years showed fair agreement, with the parental body-size classification derived from objectively measured BMI.
- After adjustment for child and parent characteristics, parental overweight and obesity were associated with lower odds of the child reporting a desire to be like the parent and with higher odds of child overweight/obesity.

**What are the implications of the main findings?**

- Young children can provide ratings of parental body size, but their limited agreement with BMI-derived classifications highlights the need for developmentally and culturally validated assessment tools.
- Children’s expressed desire to be like a parent should not be interpreted solely as desired bodily resemblance, because their responses may reflect emotional, relational, behavioural, and appearance-related considerations.

**Abstract:**

Background: Childhood obesity is a major and growing public health challenge worldwide. Although parental misperception of children’s weight is well documented, how children perceive their parents’ body size and whether parental body size is associated with children’s expressed desire to be like the parent have received little attention. We examined children’s perception of parental body size and whether parental BMI was associated with their expressed desire to be like the parent in a primary care sample. Methods: This single-center, cross-sectional study included 229 parent–child pairs consecutively recruited from a family medicine outpatient clinic in Türkiye between January and March 2026. Children aged 4–8 years rated the accompanying parent’s body size and reported whether they wanted to be like that parent, while parents independently rated their own body size. The BMI-based Body Size Guide was used for all figure selections, and the classification derived from objectively measured parental BMI served as the primary reference. Agreement was evaluated using Cohen’s kappa coefficients, associations were examined using multivariable logistic regression, and the children’s spontaneous explanations were examined using qualitative content analysis. Results: Parents showed moderate weighted agreement with the BMI-derived classification (weighted κ = 0.536, 95% CI: 0.456–0.612; exact agreement: 59.4%), whereas children showed fair weighted agreement (weighted κ = 0.315, 95% CI: 0.231–0.392; exact agreement: 42.8%). After adjustment for child age, child sex, child weight status, parent sex, and parent age, parental overweight (aOR = 0.353, 95% CI: 0.170–0.733) and parental obesity (aOR = 0.133, 95% CI: 0.057–0.307) were associated with lower odds of the child reporting a desire to be like the parent. After adjustment for child age, child sex, parent sex, and parent age, parental overweight (aOR = 2.818, 95% CI: 1.101–7.211) and parental obesity (aOR = 4.774, 95% CI: 1.816–12.546) were associated with higher odds of child overweight/obesity. Among the 53 children who provided an explanation, responses referred to body size, weight, health, appearance, affection, and identification with the parent, illustrating the broad interpretation of the question. Conclusions: Children aged 4–8 years were able to rate parental body size, although their agreement with the BMI-derived classification was limited. Children’s expressed desire to be like the parent varied according to parental BMI; however, this broad question may reflect emotional, relational, behavioural, and appearance-related considerations rather than bodily resemblance alone. The open-ended responses provide context by showing that only some children spontaneously referred to body size or weight. These findings support further research using developmentally validated and stigma-sensitive measures to clarify how young children perceive parental body size and interpret questions about identification with a parent.

## 1. Introduction

Childhood obesity is a major public health challenge. Globally, the age-standardized prevalence of obesity among school-aged children and adolescents increased from 1.7% to 6.9% in girls and from 2.1% to 9.3% in boys between 1990 and 2022 [1]. Parental underestimation of children’s weight status is common; a meta-analysis found that approximately half of parents underestimated the weight status of their children with overweight or obesity [2]. Within the family context, children are exposed to dietary and physical activity patterns as well as attitudes and communication concerning body size and weight.

Consistent with this evidence, recent studies have continued to show that parents frequently underestimate excess weight in their children [3,4]. Thus, parental perception of children’s weight has been relatively well documented. In contrast, substantially less is known about how young children perceive their parents’ body size and whether these perceptions are associated with their expressed desire to be like the parent.

Body image and body-size judgments begin to develop during early childhood. Evidence indicates that some children younger than six years can express dissatisfaction with their bodies and distinguish between socially preferred and less preferred body sizes, although estimates vary considerably according to the measurement method used [5]. Weight-related stereotypes may also emerge during the preschool years, with thinner figures often evaluated more positively than larger figures [6]. These early judgments should nevertheless be interpreted within the child’s developmental context because body-size perception, comparative judgment, and the ability to explain preferences continue to develop throughout childhood.

Figure-rating scales are commonly used to assess perceived and ideal body size because they provide a visual response format that places relatively limited demands on reading ability. However, their results may be influenced by the number, design, ordering, and visual distinguishability of the figures, as well as by the wording of the instructions and the developmental characteristics of the respondent [7,8,9]. Instruments developed to represent adult bodies may be relevant when children are asked to evaluate an adult, but validation in adults does not establish their validity for administration to children. This consideration is particularly important for children aged 4–8 years, who may differ in visual discrimination, categorization, comprehension, and verbal expression.

The development of children’s body-related attitudes takes place within a broader sociocultural context that includes parents, peers, and media. Parents may contribute through role modelling, comments about weight and appearance, eating practices, and the language used to discuss health and body size [5,10]. However, children’s responses cannot be attributed to parental influence alone, as peer interactions, media exposure, and social norms may also contribute to the development of body-size preferences [5,11].

Against this background, the present study examined how children aged 4–8 years perceive the body size of the accompanying parent and how closely their ratings correspond to the parent’s objectively measured BMI. We also compared children’s ratings with parents’ self-ratings, examined whether parental BMI was associated with the child’s expressed desire to be like the parent, and explored the reasons children gave for their responses. By focusing on children’s perceptions of their parents rather than the more commonly studied parental perception of children’s weight, this study addresses a less examined direction of body-size perception within the family.

## 2. Materials and Methods

### 2.1. Study Design

This single-center, cross-sectional study was conducted in the Family Medicine outpatient clinic of Karadeniz Technical University Faculty of Medicine between 1 January and 31 March 2026.

### 2.2. Participants and Sampling

Children aged 4–8 years who presented to the outpatient clinic for any reason during the study period accompanied by their mother or father, together with the accompanying parents, were eligible for inclusion. Exclusion criteria were the presence of a neurological or psychiatric disorder in the mother, father, or child; maternal pregnancy; and either parent’s engagement in bodybuilding sports. Neurological or psychiatric conditions were excluded because they could affect comprehension, communication, or completion of the assessment tasks. Pregnancy and bodybuilding were excluded because they may alter the relationship between BMI, body composition, and visual body shape, thereby limiting the applicability of the figure-based assessment. Information concerning neurological, psychiatric, neurodevelopmental, developmental, and speech or language disorders was obtained from the accompanying parent and checked against the available medical history and clinical records. No separate standardized neurodevelopmental or language assessment was performed for the study. Children who could not understand or communicate the assessment tasks during the individual comprehension check were not assessed further.

Children presenting with an acute condition were considered eligible only if they were clinically stable, appeared comfortable, and were able and willing to complete the assessment. If the child appeared unwell, distressed, or unable to participate because of the acute condition, the assessment was not initiated or was deferred until the child was able to participate comfortably.

Parent–child pairs meeting the eligibility criteria were recruited consecutively during the study period. No a priori sample-size calculation was performed; the study aimed to include all eligible parent–child pairs who agreed to participate during the predefined three-month recruitment period. The numbers of families assessed for eligibility, excluded before enrolment, or declining participation were not systematically recorded; therefore, a participation rate could not be calculated.

All parent–child pairs meeting the inclusion criteria and agreeing to participate were enrolled consecutively. A total of 229 parent–child pairs were reached by the end of the study period. Written informed consent was obtained from each participating parent for their own participation and from the accompanying parent or legal guardian on behalf of the child. A separate child assent form was not used.

### 2.3. Data Collection

A researcher-prepared data-collection form was used to record the child’s age and sex and the accompanying parent’s age and sex. The measured height and weight and calculated BMI values of both the child and parent were also recorded on this form. Parental education, household income, and other indicators of socioeconomic status were not collected. All child assessments were conducted by the same researcher using a standardized sequence of instructions. The purpose of the BMI-based Body Size Guide and each assessment task were explained using age-appropriate language. Before each assessment proceeded, the child was asked to explain in their own words what they were expected to do, and the researcher confirmed that the child had understood the task. No separate pilot study of the administration procedure was conducted before data collection. The child assessments were administered in the following fixed order:

(1) Selection of an ideal adult body. The child was asked to select the figure that they thought represented an ideal body size for an adult and selected one figure from the A–J Body Size Guide.

(2) Rating of the accompanying parent’s body size. The child was asked, “Which figure best represents your mother’s/father’s current body size?” and selected one figure from the sex-specific A–J guide corresponding to the accompanying parent.

(3) Selection of the body the child would like to have in adulthood. The child was asked, “Which of these figures represents the body you would like to have when you grow up?” and selected one figure from the A–J guide.

(4) Expressed desire to be like the parent. The child was asked, “When you grow up, would you like to be like your mother/father?” Response options were “yes” or “no”. The child was then invited to explain the response in their own words, and the answer was recorded verbatim.

Parents completed two figure-based assessments in the following fixed order:

(1) Parental self-rating. The parent was asked, “Which figure best represents your current body size?” and selected one figure from the sex-specific A–J Body Size Guide.

(2) Parental selection of an ideal adult body. The parent was asked, “Which figure do you think represents an ideal body size for an adult?” and selected one figure from the A–J guide.

Parents completed these assessments independently of their children.

Physician’s visual rating: A physician independently observed the accompanying parent and selected the figure that best represented the parent’s body size from the same A–J Body Size Guide. At the time of this assessment, the physician was blinded to the figure selections made by both the child and the parent and did not have access to the parent’s measured height, weight, or calculated BMI. Although the assessments were recorded consecutively on the data-collection form, each selection was made independently without knowledge of the other ratings. The physician’s selection was therefore treated as a blinded clinical visual rating and was analyzed separately from the classification derived from objectively measured BMI.

When the child attended with one parent, the accompanying parent was evaluated. When both parents were present, the parent of the same sex as the child was selected for evaluation. This a priori procedural rule was intended to provide a same-sex parent–child identification context. However, the data collection form did not separately record whether one or both parents were present; therefore, the number of cases in which this selection rule was applied could not be determined retrospectively. Child and parent assessments were conducted separately to prevent either participant from seeing or influencing the other’s responses. The accompanying parent or another familiar adult remained nearby and available to the child throughout the assessment but was positioned so that they could not see the child’s selections or influence the responses. The assessment was discontinued if the child showed discomfort or no longer wished to continue. To assess the child’s expressed desire to be like the accompanying parent, children were asked, “When you grow up, would you like to be like your mother/father?”. Response options were “yes” or “no,” after which children were invited to explain their answer in their own words. The question did not specifically refer to body size or physical appearance; therefore, it was treated as a broad measure of the desire to be like the parent rather than as a validated measure of desired bodily resemblance.

### 2.4. The BMI-Based Body Size Guide

The BMI-based Body Size Guide developed and validated by Harris et al. [12] was used to represent adult body sizes. The guide contains 10 sex-specific figures labelled A–J, arranged in order of increasing body size. Because the guide consists of visual figures labelled A–J and does not contain written questionnaire items, a separate linguistically validated Turkish version of the figure set was not used. The assessment questions and task instructions were administered in Turkish using standardized wording prepared by the researchers; however, this wording had not undergone a separate formal linguistic validation process. The adult figures were considered relevant to the target of the assessment because the children were asked to evaluate an adult parent. Nevertheless, the cultural appropriateness of the figures and their validity when used by Turkish children aged 4–8 years have not been established. The figures correspond to the following BMI ranges: A, underweight (<18.5 kg/m^2^); B–C, normal weight (18.5–24.9 kg/m^2^); D, overweight (25.0–29.9 kg/m^2^); E–F, obesity class I (30.0–34.9 kg/m^2^); G–H, obesity class II (35.0–39.9 kg/m^2^); and I–J, obesity class III (≥40.0 kg/m^2^). For the four-category analyses, figures E–J were combined into a single obesity category. The BMI-derived reference classification was determined from the parent’s objectively measured height and weight and was not based on the physician’s figure selection. The physician independently selected an A–J figure before the anthropometric measurements were available, and this selection was analyzed separately as a blinded visual rating. Although the guide was developed to represent adult bodies, its original validation in adults does not establish its validity for administration to children aged 4–8 years.

### 2.5. Anthropometric Measurements and BMI Assessment

All height and weight measurements were performed by the researchers using standardized stadiometers and scales. BMI was calculated as weight (kg)/height^2^ (m^2^). Parental BMI was classified according to World Health Organization criteria as underweight (<18.5), normal weight (18.5–24.9), overweight (25.0–29.9), or obesity (≥30.0). Children’s BMI was evaluated using age- and sex-specific BMI percentile reference values for Turkish children [13]. Children were classified as underweight (<5th percentile), normal weight (≥5th to <85th percentile), overweight (≥85th to <95th percentile), or having obesity (≥95th percentile). For the multivariable analyses, child weight status was dichotomized as overweight/obesity versus underweight/normal weight. Parental BMI was calculated from the objectively measured height and weight and classified as underweight, normal weight, overweight, or obesity. This BMI-derived four-category classification constituted the primary reference for evaluating parental self-ratings and children’s ratings of parental body size. The physician’s blinded A–J figure selection was analyzed separately as a secondary clinical visual rating.

### 2.6. Statistical Analysis

Data were analyzed using SPSS version 27.0 (IBM Corp., Armonk, NY, USA). Descriptive statistics are presented as number and percentage for categorical variables, and as mean ± standard deviation, minimum, and maximum for numerical variables. There were no missing data for the quantitative variables included in the primary agreement analyses or multivariable models; therefore, no statistical imputation was performed. Open-ended explanations were optional, and blank responses were treated as non-response and excluded only from the qualitative content analysis.

Agreement of parental self-ratings and children’s ratings of parental body size with the BMI-derived four-category classification was examined as the primary analysis of perceptual accuracy. Agreement with the physician’s blinded visual rating was examined separately as a secondary analysis. For the secondary figure-level analysis, selection of the same A–J figure as the physician or an immediately adjacent figure was classified as ±1-figure near agreement. Because the four weight-status categories were ordinal, both unweighted and linearly weighted Cohen’s kappa coefficients were calculated. Linear weighting assigns less weight to disagreement between adjacent categories and progressively greater weight to disagreement between more distant categories. Ninety-five percent confidence intervals were calculated for all kappa coefficients. The strength of agreement was interpreted using the following thresholds: ≤0.20, slight; 0.21–0.40, fair; 0.41–0.60, moderate; 0.61–0.80, substantial; and 0.81–1.00, almost perfect. Interpretation of the strength of agreement was based primarily on the linearly weighted kappa coefficients.

Perceptual agreement was evaluated at two complementary levels. First, category-level accuracy was defined as agreement between the BMI category represented by the selected figure and the parent’s objectively determined BMI category, using the four-category classification of underweight, normal weight, overweight, and obesity. Second, figure-level near agreement was evaluated on the 10-figure A–J continuum. In this analysis, selection of the same A–J figure as the physician’s blinded visual rating or an immediately adjacent figure was accepted as ±1-figure near agreement. For example, when the physician selected figure E, selections of D, E, or F met this criterion. The two analyses were reported separately because they assess clinically interpretable BMI-category accuracy and fine-grained visual proximity, respectively.

Associations between categorical variables were analyzed using Pearson’s chi-square test, Fisher’s exact test, or the Fisher–Freeman–Halton test, as appropriate. Two multivariable binary logistic regression models were constructed. In the first model, the child’s expressed desire to be like the parent was the dependent variable, with “yes” coded as 1 and “no” as 0. Parental BMI category was the principal independent variable, and the model was adjusted for child age, child sex, child overweight/obesity status, the sex of the evaluated parent, and parent age. In the second model, child overweight/obesity was the dependent variable, with overweight or obesity coded as 1 and underweight or normal weight as 0. This model included parental BMI category, child age, child sex, the sex of the evaluated parent, and parent age. Underweight and normal-weight parents were combined as the reference category because of the small number of underweight parents. Child age and parent age were entered as continuous variables. Child sex and parent sex were entered as separate covariates despite their association arising from the parent-selection procedure. Collinearity diagnostics did not indicate problematic multicollinearity. Adjusted odds ratios (aORs), 95% confidence intervals (CIs), and *p* values were reported. Children’s brief open-ended explanations for their responses to the question “When you grow up, would you like to be like your mother/father?” were examined using inductive qualitative content analysis. Blank responses were treated as non-response and were not included in the qualitative analysis. The coding team consisted of a family medicine physician who had conducted the participant assessments and a public health specialist who had not been involved in participant recruitment or data collection. The two researchers independently reviewed the responses and developed preliminary codes directly from the data. Differences in coding were resolved through discussion and consensus. Because most responses consisted of a single brief statement expressing one principal idea, one primary content category was assigned to each response, and multiple coding was not used. A formal intercoder reliability coefficient was not calculated. Absolute frequencies and percentages were calculated separately for children who answered “yes” and “no,” using the number of children providing an explanation in the respective response group as the denominator. Characteristics of children who did and did not provide an explanation were compared using chi-square or Fisher’s exact tests, as appropriate. The level of statistical significance was set at *p* < 0.05.

## 3. Results

### 3.1. Participant Characteristics

**Figure 1:**
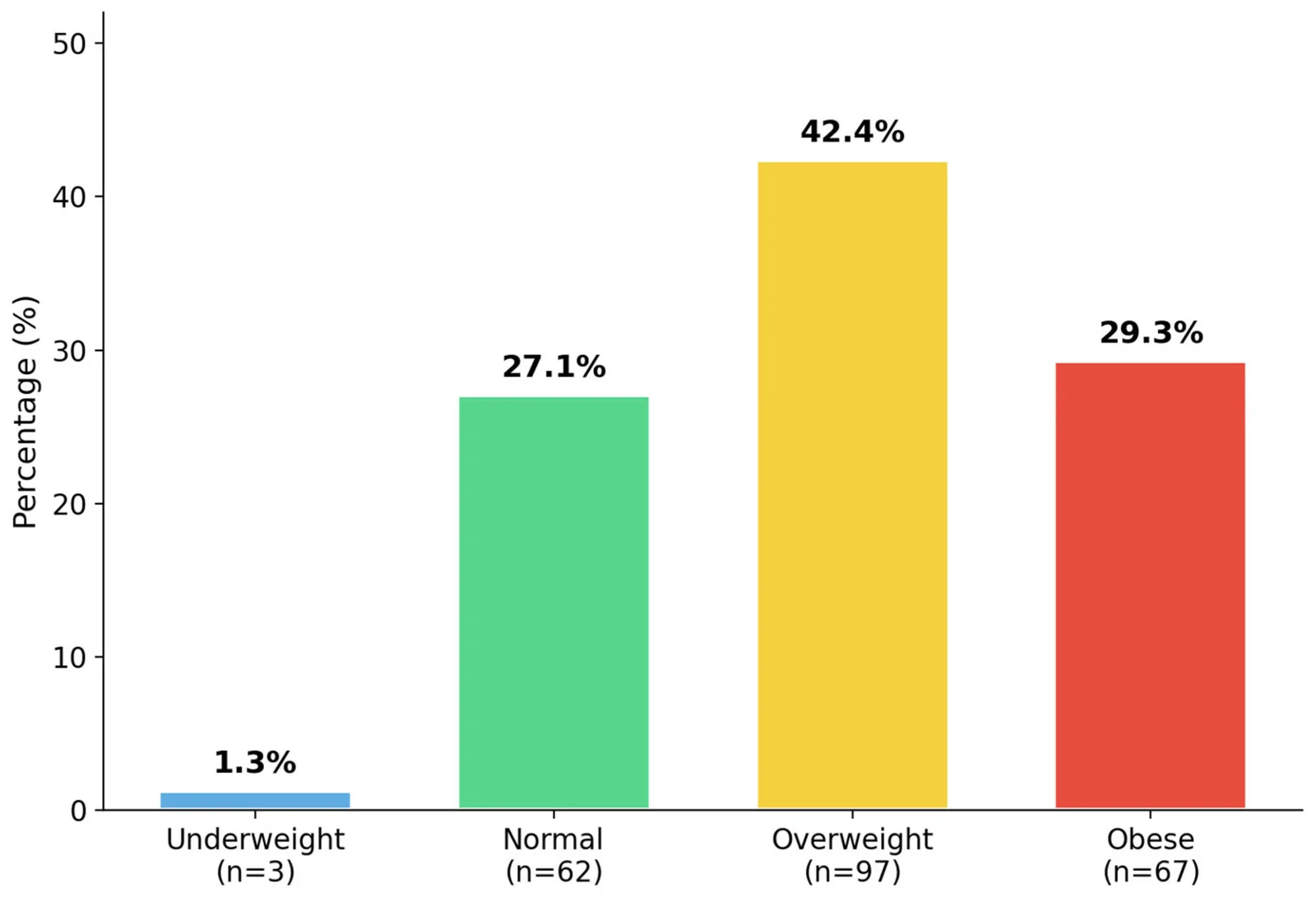
Distribution of parents by BMI category. A total of 71.7% of parents were classified as overweight or obese.

A total of 229 parent–child pairs were included in the study. Because the numbers of families assessed for eligibility, excluded before enrolment, or declining participation were not systematically recorded, a complete participant flow and participation rate could not be reported. Of the parents, 74.2% (*n* = 170) were mothers and 25.8% (*n* = 59) were fathers. The mean age of parents was 38.24 ± 5.93 years and the mean BMI was 27.76 ± 4.76 kg/m^2^. According to BMI classification, 1.3% of parents were underweight, 27.1% normal weight, 42.4% overweight, and 29.3% obese (Figure 1).

Among the children, 66.4% (*n* = 152) were girls and 33.6% (*n* = 77) were boys. Among the 77 boys, the evaluated parent was the father in 53 cases (68.8%) and the mother in 24 cases (31.2%). Among the 152 girls, the evaluated parent was the mother in 146 cases (96.1%) and the father in six cases (3.9%). The mean age was 6.30 ± 1.36 years (range 4–8 years) and the mean BMI was 16.44 ± 2.97 kg/m^2^. According to the age- and sex-specific BMI percentiles, 23 children (10.0%) were underweight, 149 (65.1%) had normal weight, 31 (13.5%) were overweight, and 26 (11.4%) had obesity (Table 1).

### 3.2. Parental Self-Perception of Body Size

**Figure 2:**
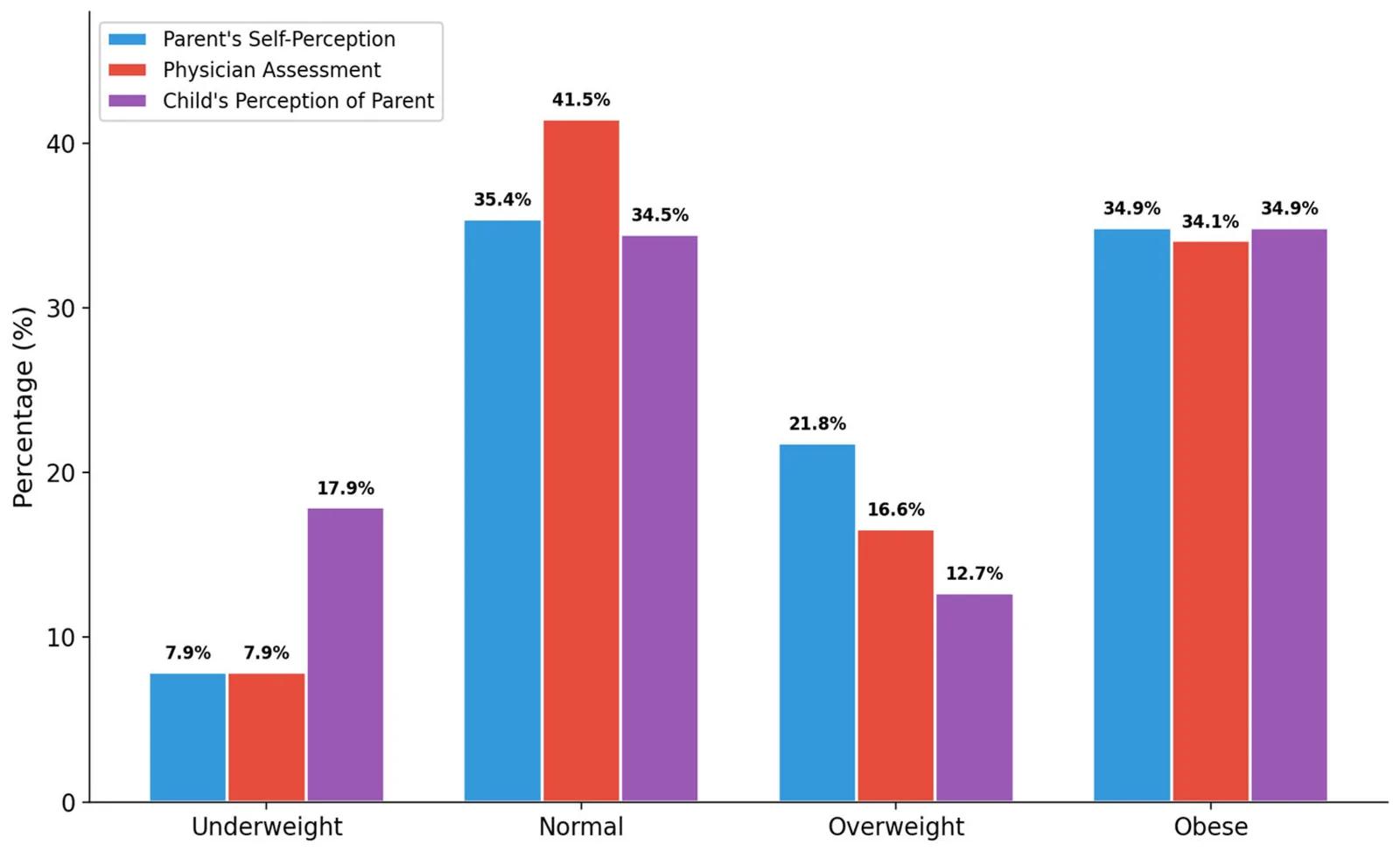
Distribution of body-size ratings across parental self-ratings, the physician’s blinded visual ratings, and children’s ratings of the parent.

In the primary analysis using the BMI-derived classification as the objective reference, exact four-category agreement was observed in 59.4% of parental self-ratings. The unweighted kappa coefficient was 0.427 (95% CI: 0.342–0.513), while the linearly weighted kappa indicated moderate agreement (weighted κ = 0.536, 95% CI: 0.456–0.612; *p* < 0.001). In the secondary analysis, parental self-ratings showed 58.5% exact four-category agreement with the physician’s blinded visual rating. The corresponding unweighted kappa was 0.400 (95% CI: 0.307–0.495), and the linearly weighted kappa indicated moderate agreement (weighted κ = 0.554, 95% CI: 0.470–0.633; *p* < 0.001). On the A–J continuum, 80.3% of parental self-ratings were either identical to or immediately adjacent to the physician’s selected figure (Figure 2).

### 3.3. Children’s Perception of Parental Body Size

In the primary analysis using the BMI-derived classification as the objective reference, exact four-category agreement was observed in 42.8% of children’s ratings of parental body size. The unweighted kappa coefficient was 0.236 (95% CI: 0.159–0.311), while the linearly weighted kappa indicated fair agreement (weighted κ = 0.315, 95% CI: 0.231–0.392; *p* < 0.001). In the secondary analysis, children’s ratings showed 48.0% exact four-category agreement with the physician’s blinded visual rating. The corresponding unweighted kappa was 0.261 (95% CI: 0.175–0.340), and the linearly weighted kappa also indicated fair agreement (weighted κ = 0.350, 95% CI: 0.249–0.441; *p* < 0.001). On the A–J continuum, 58.1% of children selected either the same figure as the physician or an immediately adjacent figure (Table 2).

**Figure 3:**
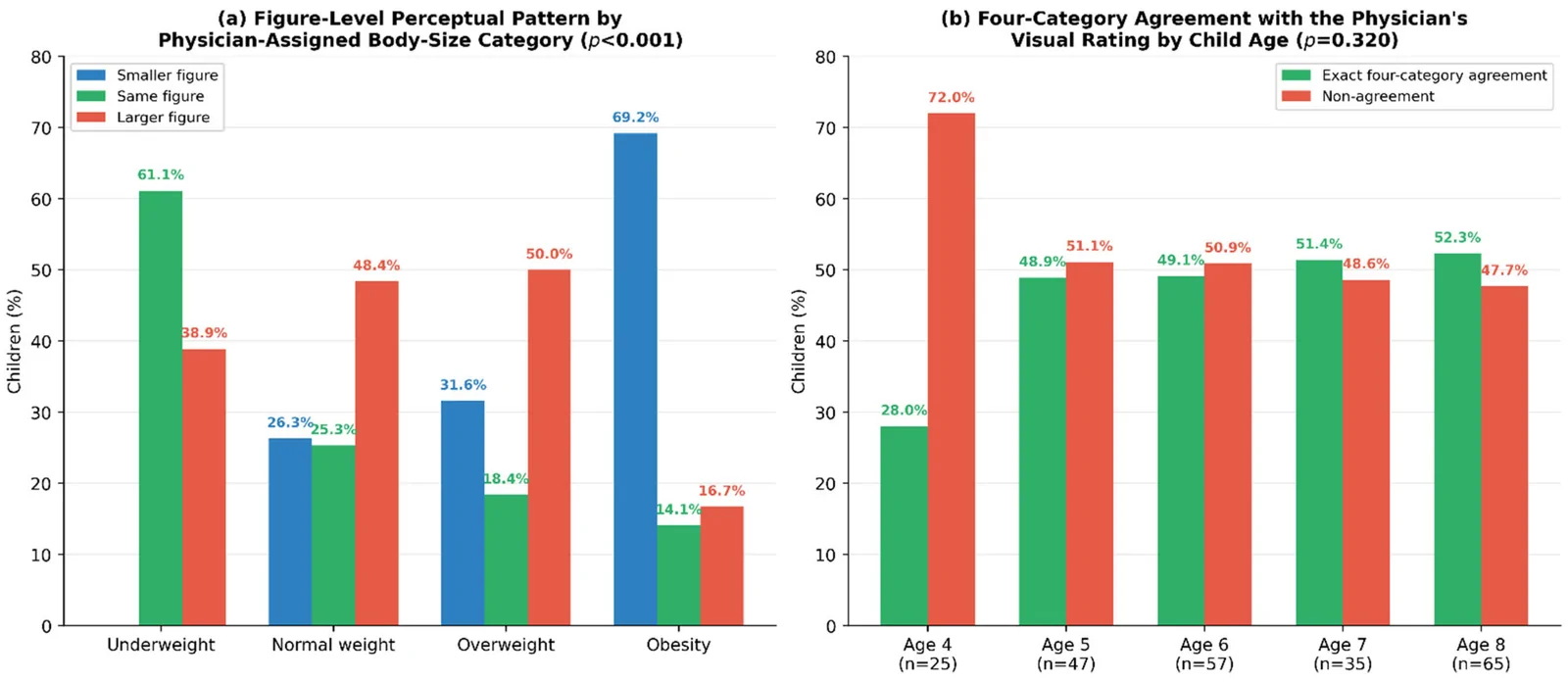
Children’s perception of parental body size in relation to the physician’s blinded visual rating: (**a**) figure-level perceptual pattern according to the physician-assigned body-size category; (**b**) exact four-category agreement between children’s ratings and the physician’s visual rating according to child age.

Figure-level perceptual patterns differed across the physician-assigned body-size categories (*p* < 0.001). Among children whose parents were assigned an underweight-range figure by the physician, 61.1% selected the same A–J figure as the physician. In contrast, among children whose parents were assigned an obesity-range figure, 14.1% selected the same figure and 69.2% selected a smaller figure. Four-category agreement with the physician’s blinded visual rating increased from 28.0% at age 4 to 52.3% at age 8, but the difference was not statistically significant (*p* = 0.320; Figure 3).

### 3.4. Children’s Desire to Be Like the Parent

**Figure 4:**
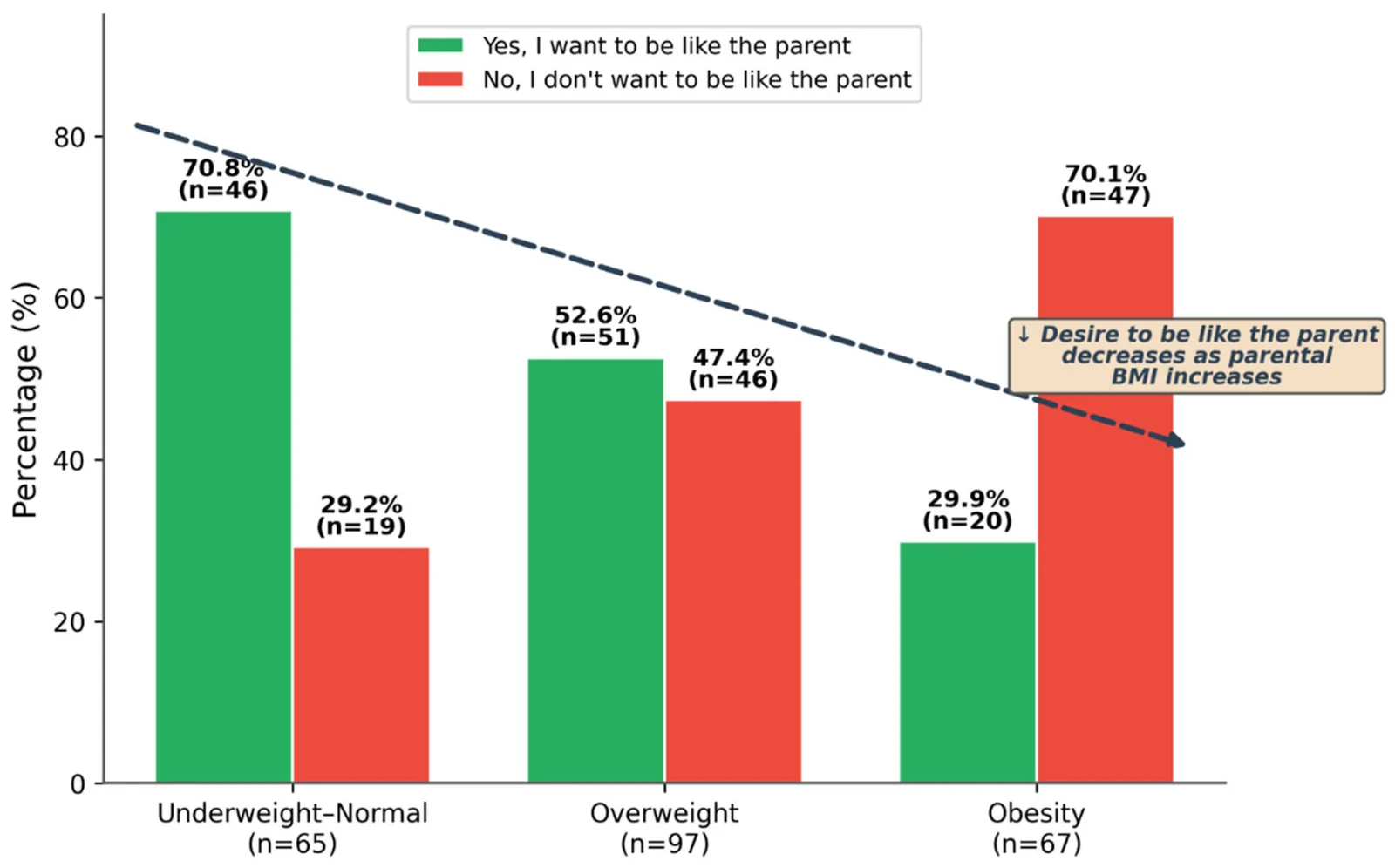
Children’s expressed desire to be like the parent according to parental BMI category.

Of the children, 51.1% (*n* = 117) reported that they would like to be like the accompanying parent when they grew up, whereas 48.9% (*n* = 112) responded that they would not. The distribution of responses differed across parental BMI categories (*p* < 0.001). The proportion responding “yes” was 70.8% among children of underweight or normal-weight parents, 52.6% among children of overweight parents, and 29.9% among children of parents with obesity (Figure 4). A similar pattern was observed according to the physician’s blinded visual classification: the proportion responding “yes” was 72.2% in the underweight category, 62.1% in the normal-weight category, 52.6% in the overweight category, and 32.1% in the obesity category (*p* < 0.001).

### 3.5. Parental BMI and Child Obesity

Parental BMI category was associated with child weight status (*p* = 0.033). The prevalence of overweight/obesity was 10.8% among children of underweight or normal-weight parents, 27.8% among children of overweight parents, and 34.3% among children of parents with obesity.

### 3.6. Multivariable Analyses

In the multivariable model examining the child’s expressed desire to be like the parent, parental BMI remained associated with the response after adjustment for child age, child sex, child overweight/obesity status, parent sex, and parent age. Compared with children of underweight or normal-weight parents, children of overweight parents had lower odds of reporting that they wanted to be like the parent (aOR = 0.353, 95% CI: 0.170–0.733; *p* = 0.005). The corresponding odds were also lower among children of parents with obesity (aOR = 0.133, 95% CI: 0.057–0.307; *p* < 0.001). Increasing parent age was associated with lower odds of a “yes” response (aOR = 0.911 per year, 95% CI: 0.861–0.964; *p* = 0.001). Child age, child sex, child overweight/obesity status, and parent sex were not statistically significant in the adjusted model (Table 3).

In the model examining child overweight/obesity, children of overweight parents had higher odds of overweight/obesity (aOR = 2.818, 95% CI: 1.101–7.211; *p* = 0.031), as did children of parents with obesity (aOR = 4.774, 95% CI: 1.816–12.546; *p* = 0.002), compared with children of underweight or normal-weight parents. Each one-year increase in child age was associated with higher odds of overweight/obesity (aOR = 1.535, 95% CI: 1.175–2.004; *p* = 0.002). Child sex, parent sex, and parent age were not statistically significant (Table 4).

### 3.7. Ideal Adult Body and Desired Future Body

**Figure 5:**
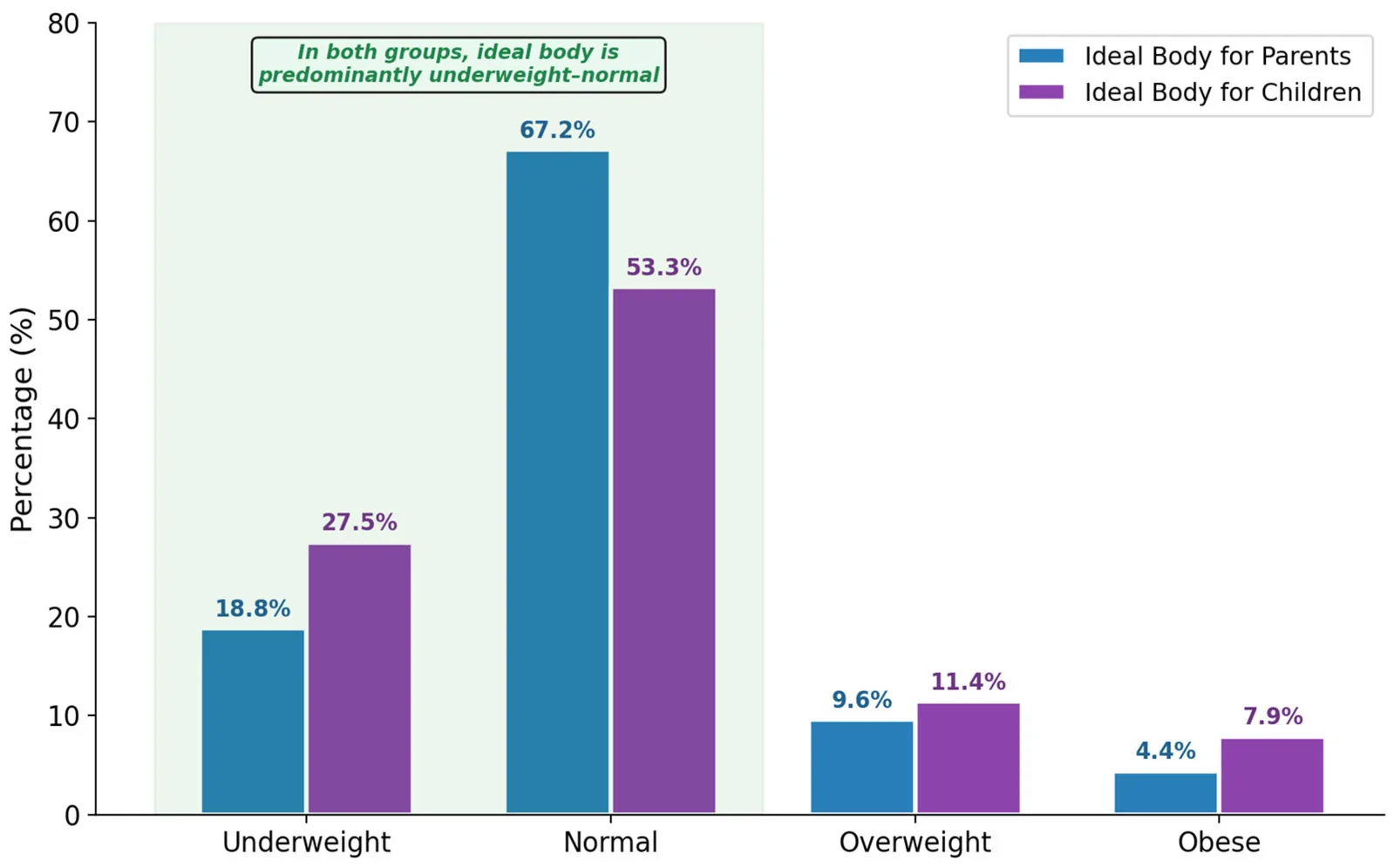
Parent and child selections of an ideal adult body according to the four-category weight classification.

A total of 86.0% of parents selected an underweight or normal-weight figure as the ideal adult body, while 80.8% of children selected a figure within the same categories as the ideal adult body (Figure 5). Exact four-category agreement between children’s selection of an ideal adult body and their desired future body was 63.8%. The linearly weighted kappa indicated moderate agreement (weighted κ = 0.432, 95% CI: 0.327–0.535; *p* < 0.001). No statistically significant association was found between the parent’s BMI-derived category and the child’s desired future body (*p* = 0.350). Similarly, no statistically significant agreement was found between the physician’s blinded visual classification of parental body size and the child’s desired future body (κ = 0.039, *p* = 0.258).

### 3.8. Comparison of Ratings with the Physician’s Blinded Visual Rating

**Figure 6:**
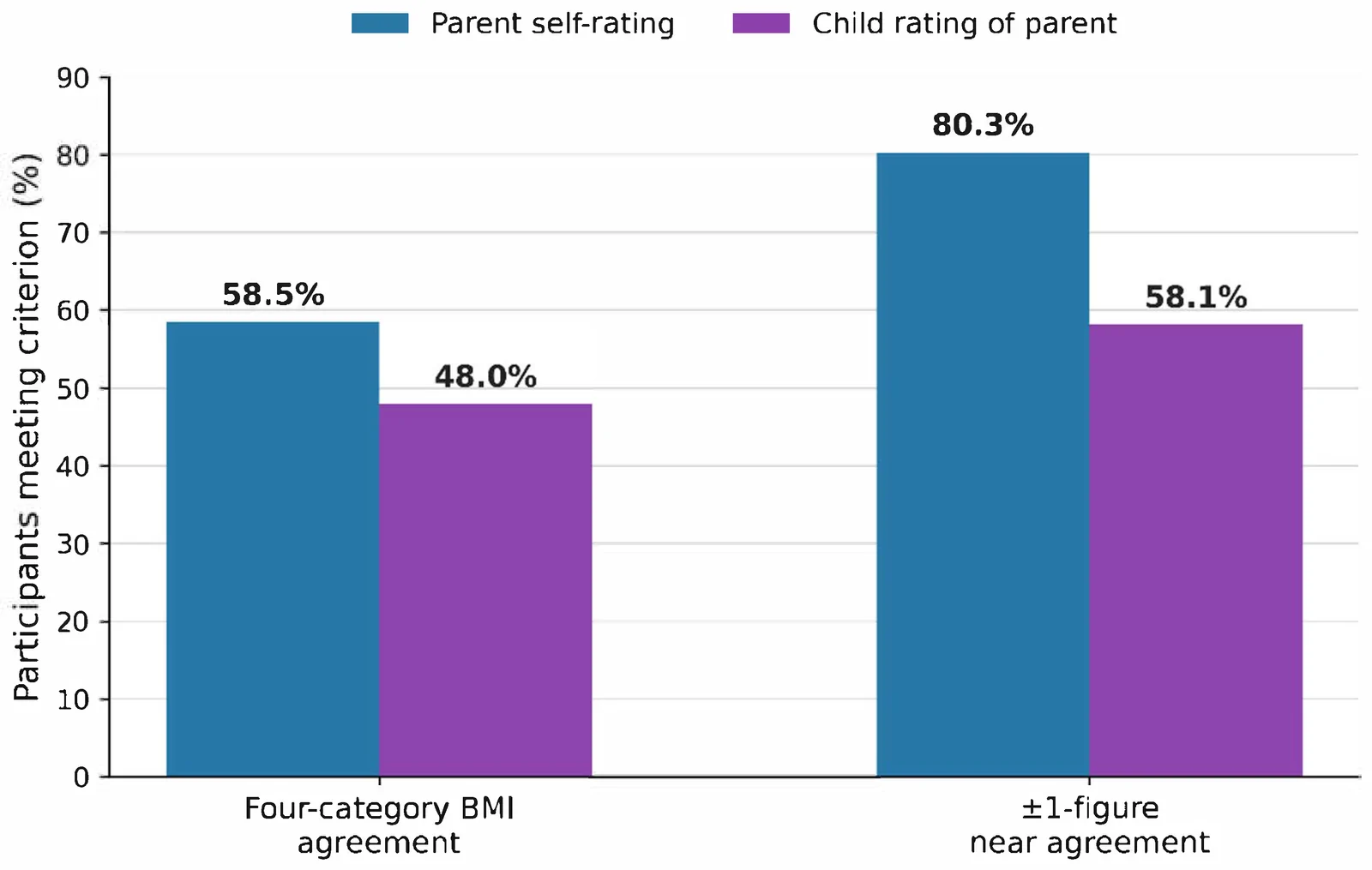
Comparison of exact four-category agreement with the physician’s blinded visual rating and ±1-figure near agreement on the A–J continuum for parent self-ratings and children’s ratings of their parents.

On the A–J continuum, ±1-figure near agreement with the physician’s blinded visual rating was observed in 80.3% of parental self-ratings and 58.1% of children’s ratings. Exact four-category agreement with the physician’s blinded visual rating was 58.5% for parental self-ratings and 48.0% for children’s ratings. The corresponding unweighted kappa coefficients were 0.400 (95% CI: 0.307–0.495) for parental self-ratings and 0.261 (95% CI: 0.175–0.340) for children’s ratings (Figure 6).

### 3.9. Qualitative Findings

Children were asked, “When you grow up, would you like to be like your mother/father?” and were invited to explain their response in their own words. Because the question did not specifically mention body size or appearance, the qualitative analysis was used to examine the types of reasons children spontaneously provided. Of the 229 children, 53 (23.1%) provided an explanation for their response, whereas 176 (76.9%) did not. Explanations were provided by 27 of the 117 children who reported wanting to be like the parent (23.1%) and by 26 of the 112 children who did not (23.2%). The proportion providing an explanation did not differ significantly according to child age (*p* = 0.546), child sex (*p* = 0.661), child weight status (*p* = 0.911), parent sex (*p* = 0.509), parental BMI category (*p* = 0.223), or the child’s yes/no response to the question (*p* = 1.000).

Among the 27 children who reported wanting to be like the parent and provided an explanation, 17 responses (63.0%) referred primarily to body size, weight, strength, health, or weight-related norms. These included statements about being thinner or stronger, having a better body, avoiding overweight, and the parent having an ideal or normal weight. Seven responses (25.9%) reflected appearance-related approval, general admiration, or identification with the parent, such as describing the parent as beautiful or handsome or expressing a wish to resemble the parent. The remaining three responses (11.1%) were unclear, broadly negative, or inconsistent with the yes/no response.

Among the 26 children who reported not wanting to be like the parent and provided an explanation, 14 responses (53.8%) referred primarily to body size, weight, health, or weight-related norms, including the parent being overweight or too thin, the child wanting to be thinner, and references to health, healthy eating, or ideal weight. Eleven responses (42.3%) nevertheless expressed affection, appearance-related approval, or admiration for the parent. One response (3.8%) expressed a general desire to be different. The presence of positive or affectionate explanations among some children who answered “no,” as well as critical body-related explanations among some who answered “yes,” illustrates the broad and potentially ambiguous interpretation of the question.

## 4. Discussion

This study examined children’s and parents’ ratings of parental body size and the associations of parental BMI with the child’s expressed desire to be like the parent and child weight status. Parents showed moderate agreement and children showed fair agreement with the parental BMI-derived classification. Parental overweight and obesity were associated with lower odds of the child reporting a desire to be like the parent and with higher odds of child overweight/obesity in the adjusted analyses. No statistically significant association was observed between parental BMI category and the child’s desired future body.

Parental self-ratings showed moderate agreement with the BMI-derived classification. In the secondary comparison with the physician’s blinded visual rating, 80.3% of parental self-ratings were either identical to or immediately adjacent to the physician’s selected figure. This represents figure-level near agreement and should not be interpreted as exact category-level accuracy. Errors in self-evaluation of body size have also been reported in previous studies [7]. Visual normalization has been proposed as one potential explanation for body-size underestimation in populations with a high prevalence of overweight and obesity [14]. Although 71.7% of the parents in our sample were overweight or had obesity, normalization processes were not measured; therefore, this mechanism cannot be established from the present findings.

Children’s ratings showed fair agreement with the parental BMI-derived classification. In the secondary comparison with the physician’s blinded visual rating, 58.1% of children selected either the same A–J figure as the physician or an immediately adjacent figure. This figure-level near-agreement rate was lower than that observed for parental self-ratings. Developmental differences in visual discrimination, categorization, and comparative judgment may have contributed to this pattern. Previous research suggests that body-size perception may not be fully developed even among children aged 7–9 years [15]. However, these developmental abilities were not measured directly, and the Body Size Guide has not been validated for administration to Turkish children aged 4–8 years. The comparison should therefore be interpreted cautiously.

Among children whose parents were assigned an obesity-range figure by the physician, 69.2% selected a smaller figure than the physician (*p* < 0.001). This pattern may reflect children’s familiarity with frequently encountered body sizes, developmental limitations in visual discrimination, or other unmeasured influences. Although visual normalization offers one possible explanation [14], normalization processes were not directly assessed, and the finding should not be interpreted as evidence of a demonstrated mechanism. Four-category agreement with the physician’s visual rating increased from 28.0% at age 4 to 52.3% at age 8; however, this difference was not statistically significant (*p* = 0.320). Therefore, no conclusion can be drawn regarding age-related maturation of body-size perception, and this possibility should be examined in larger developmental studies.

The proportion of children who expressed a desire to be like the accompanying parent decreased across increasing parental BMI categories. After adjustment for child age, child sex, child overweight/obesity status, parent sex, and parent age, parental overweight (aOR = 0.353, 95% CI: 0.170–0.733) and parental obesity (aOR = 0.133, 95% CI: 0.057–0.307) were associated with lower odds of the child reporting a desire to be like the parent. However, this association should not be interpreted solely in terms of desired bodily resemblance. The question was broad and may have reflected affection, identification, admiration, personality, behaviour, parental role, physical appearance, or a combination of these considerations. Moreover, because the study was cross-sectional, the direction and underlying mechanisms of the association cannot be determined. Previous research suggests that body-size stereotypes may emerge during the preschool years [6], but the present study did not directly assess weight bias or stigma.

Parental BMI was also associated with child weight status. After adjustment for child age, child sex, parent sex, and parent age, children of overweight parents had higher odds of overweight/obesity (aOR = 2.818, 95% CI: 1.101–7.211), as did children of parents with obesity (aOR = 4.774, 95% CI: 1.816–12.546), compared with children of underweight or normal-weight parents. Increasing child age was also associated with higher odds of overweight/obesity (aOR = 1.535 per year, 95% CI: 1.175–2.004). The coexistence of lower odds of reporting a desire to be like a parent with obesity and higher odds of overweight/obesity among these children suggests that the child’s expressed identification with the parent does not necessarily correspond to the child’s weight status. This pattern is consistent with the multifactorial nature of childhood obesity, in which genetic, behavioural, and shared environmental factors may contribute [16]; however, these factors were not directly assessed in the present study.

The majority of both parents (86.0%) and children (80.8%) defined the ideal body as underweight or normal weight. Moderate agreement was found between children’s selection of an ideal adult body and the body they reported wanting to have in adulthood (linearly weighted κ = 0.432, 95% CI: 0.327–0.535). In this sample, no statistically significant association was found between parental BMI category and the child’s desired future body. The absence of a statistically significant association does not establish that children’s desired future bodies were independent of parental BMI or parental influence. Previous studies suggest that media exposure, peer interactions, and broader social norms may also contribute to body-related preferences during childhood [11]; however, these factors were not measured in the present study.

Systematic reviews have shown that overweight and obesity during childhood frequently persist into adulthood [17,18]. These longitudinal risks underscore the importance of childhood obesity prevention. Family involvement is recommended in the assessment and treatment of childhood obesity, and family-based interventions may improve some health behaviours [19,20]. However, the present study did not evaluate an intervention, and it did not directly assess dietary patterns, physical activity, genetic influences, normalization processes, or family weight-related communication. Therefore, the observed associations cannot identify the mechanisms linking parental and child weight status and do not demonstrate that incorporating body-perception assessment would improve intervention effectiveness. The findings instead identify questions that may be examined in future prospective and intervention studies.

The brief open-ended explanations help clarify the conceptual breadth of the question. Body size, weight, health, and weight-related norms appeared in the explanations of children who both did and did not want to be like the parent. Appearance-related approval, affection, and admiration were also expressed in both groups. These brief statements cannot distinguish whether body- or weight-related expressions reflected personal body preferences, health beliefs, social norms, or weight stigma. Moreover, some explanations appeared inconsistent with the preceding yes/no response. These findings reinforce that the question was not interpreted exclusively in terms of bodily resemblance and may have reflected emotional attachment, admiration, appearance, health beliefs, or general identification with the parent. Because only 53 children provided an explanation and the responses were generally limited to a single brief statement, these findings should be regarded as descriptive contextual information rather than an in-depth qualitative account.

## 5. Limitations

Our study has several limitations. First, because of the cross-sectional design, temporal ordering and causal relationships among parental BMI, children’s perception of parental body size, the child’s expressed desire to be like the parent, and child weight status cannot be established. Although the multivariable models included child age, child sex, child weight status where applicable, parent sex, and parent age, residual confounding remains possible. Socioeconomic status, parental education, dietary patterns, physical activity, media exposure, and family weight-related communication were not measured and could not be included in the analyses. Second, the study used consecutive recruitment in a single family-medicine clinic, which limits the representativeness and generalizability of the findings. Because a screening log was not maintained, the numbers of families assessed for eligibility, excluded, or declining participation could not be reported, and potential non-participation bias could not be evaluated. No a priori sample-size calculation was performed, and the sample size was determined by the number of eligible parent–child pairs enrolled during the predefined study period. The very small number of underweight parents also limited separate subgroup analyses; therefore, underweight and normal-weight parents were combined in the multivariable models. Third, the Body Size Guide was originally developed and validated in adults and has not been psychometrically validated for administration to Turkish children aged 4–8 years. Its cultural appropriateness for this population and the researcher-prepared Turkish instructions have also not been formally evaluated. Although standardized, age-appropriate instructions and an individual comprehension check were used, these procedures do not establish the validity or reliability of the instrument in this age group. In addition, no separate pilot study was conducted before data collection. Body-size perception may not yet be fully developed in younger children [11], and children across the 4–8-year age range may differ substantially in visual discrimination, categorization, language comprehension, comparative judgment, and understanding of concepts such as an “ideal adult body”. These developmental and measurement-related factors may have influenced the figure selections, particularly among the youngest children, and the findings should therefore be interpreted cautiously. In addition, the absence of neurological, psychiatric, neurodevelopmental, or speech and language disorders was determined from parental report and available clinical information rather than through a standardized developmental assessment. Therefore, unrecognized developmental or communication difficulties cannot be excluded. Fourth, the question “When you grow up, would you like to be like your mother/father?” was broad and was not a validated measure of desired physical or bodily resemblance. Children may have interpreted the question in relation to affection, admiration, personality, behaviour, parental role, physical appearance, or a combination of these characteristics. Therefore, the association between parental BMI and the child’s response should not be interpreted as evidence that parental body size determined the child’s desire to be like the parent. Although the open-ended responses provided contextual information, they do not fully resolve this conceptual ambiguity. In addition, the child assessments were administered in a fixed order rather than being randomized or counterbalanced. Children selected an ideal adult body, rated the accompanying parent’s body size, and selected their desired future body before being asked whether they wanted to be like the parent and why. This sequence may have increased the salience of body size and weight and may have influenced both the yes/no response and the reasons subsequently provided. Therefore, a priming or order effect cannot be excluded. Fifth, the sex distributions of both the children and the evaluated parents were unbalanced: girls constituted 66.4% of the child sample, while mothers constituted 74.2% of the evaluated parents. In addition, when both parents were present, the parent of the same sex as the child was selected. Because the data collection form did not record whether one or both parents attended, the number of cases in which this rule was applied could not be determined retrospectively. This procedure may have created or strengthened an association between child sex and parent sex and may therefore have introduced selection-related bias. Although child sex and parent sex were considered separately in the multivariable analyses, their independent effects may be difficult to distinguish because of the strong correspondence between these variables. The findings concerning parent-sex and child-sex differences should therefore be interpreted cautiously. Sixth, only 53 of the 229 children (23.1%) provided an explanation for their yes/no response. Although children who did and did not provide an explanation did not differ significantly in the measured characteristics examined, non-response bias related to unmeasured characteristics cannot be excluded. The responses were brief and usually consisted of a single statement; therefore, the qualitative content analysis was limited in depth and should be interpreted as descriptive and exploratory. The small numbers within individual content categories also precluded detailed subgroup comparisons.

## 6. Conclusions

This study found limited agreement between children’s ratings of parental body size and the parental BMI-derived classification. Parental overweight and obesity were independently associated with lower odds of the child reporting a desire to be like the parent and with higher odds of child overweight/obesity. However, the broad and non-validated nature of the “be like the parent” question, the cross-sectional design, and the developmental limitations of the figure-rating task preclude causal or specifically body-related interpretations. The findings should therefore be considered hypothesis-generating. Further studies using developmentally validated and stigma-sensitive measures are needed to assess the feasibility, acceptability, and potential value of examining parent–child body-size perception in family-based care before any routine clinical application can be recommended.

## Figures and Tables

**Table 1:** Sociodemographic and anthropometric characteristics of participants (*n* = 229).

Variable	Mean	SD	Min	Max
Parent age (years)	38.24	5.93	24	60
Parent height (cm)	164.19	8.69	148	193
Parent weight (kg)	75.10	15.21	40	120
Parent BMI (kg/m^2^)	27.76	4.76	17.04	44.54
Child age (years)	6.30	1.36	4	8
Child height (cm)	120.40	10.41	94	152
Child weight (kg)	24.33	7.81	12	60
Child BMI (kg/m^2^)	16.44	2.97	10.29	28.53

BMI, body mass index; SD, standard deviation.

**Table 2:** Agreement of parental and child ratings with the BMI-derived classification and the physician’s blinded visual rating.

Comparison	Exact Agreement (%)	Unweighted κ (95% CI)	Linearly Weighted κ (95% CI)	Weighted Agreement	*p*
Parent self-rating vs. BMI-derived classification	59.4	0.427 (0.342–0.513)	0.536 (0.456–0.612)	Moderate	<0.001
Child rating of parent vs. BMI-derived classification	42.8	0.236 (0.159–0.311)	0.315 (0.231–0.392)	Fair	<0.001
Parent self-rating vs. physician’s blinded visual rating	58.5	0.400 (0.307–0.495)	0.554 (0.470–0.633)	Moderate	<0.001
Child rating of parent vs. physician’s blinded visual rating	48.0	0.261 (0.175–0.340)	0.350 (0.249–0.441)	Fair	<0.001

BMI, body mass index; CI, confidence interval; κ, Cohen’s kappa coefficient. Agreement was interpreted as fair (0.21–0.40) or moderate (0.41–0.60) according to standard cutoffs.

**Table 3:** Multivariable logistic regression analyses of children’s desire to be like the parent.

Variable	aOR	95% CI	*p*
Parental BMI category			
Underweight/normal weight	1	Ref.	
Overweight	0.353	0.170–0.733	0.005
Obesity	0.133	0.057–0.307	<0.001
Child age, per year	0.833	0.662–1.048	0.118
Female child	0.738	0.306–1.782	0.500
Child overweight/obesity	0.648	0.322–1.304	0.224
Female parent	0.493	0.188–1.291	0.150
Parent age, per year	0.911	0.861–0.964	0.001

aOR, adjusted odds ratio; BMI, body mass index; CI, confidence interval.

**Table 4:** Multivariable logistic regression analyses of child overweight/obesity.

Variable	aOR	95% CI	*p*
Parental BMI category			
Underweight/normal weight	1	Ref.	
Overweight	2.818	1.101–7.211	0.031
Obesity	4.774	1.816–12.546	0.002
Child age, per year	1.535	1.175–2.004	0.002
Female child	0.870	0.335–2.263	0.776
Female parent	0.686	0.252–1.870	0.462
Parent age, per year	0.979	0.927–1.035	0.462

aOR, adjusted odds ratio; BMI, body mass index; CI, confidence interval.

## Data Availability

The datasets generated and analyzed during the current study are not publicly available due to participant privacy considerations but are available from the corresponding author on reasonable request.
